# Submassive Pulmonary Embolism Complicating Small Bowel Obstruction

**DOI:** 10.7759/cureus.17162

**Published:** 2021-08-13

**Authors:** Andrew Sagalov, Sorcha Allen, Omer Iqbal, Amir Darki

**Affiliations:** 1 Internal Medicine, Arkansas College of Osteopathic Medicine, Fort Smith, USA; 2 Interventional Cardiology, Loyola University Medical Center, Maywood, USA; 3 Pathology, Loyola University Medical Center, Maywood, USA

**Keywords:** pulmonary embolism (pe), small bowel obstruction, anticoagulation, pulmonary embolism response team (pert), hemodynamics

## Abstract

The evaluation of patients with pulmonary embolism (PE) requires a comprehensive approach that assesses physical and laboratory exam findings, interprets varying imaging modalities, and selects appropriate treatment strategies. Comorbidities can complicate treatment and influence physicians to make difficult management decisions. In this case report, we present a patient with a small bowel obstruction complicated by submassive intermediate high-risk PE. Stratifying risk in PE patients is crucial to gauge when to use invasive interventions. There is limited clinical data to identify the optimal timing of surgery in patients with concurrent PE. We describe a challenging case where a patient requires multiple life-saving interventions; however, each treatment method carries a risk of bleeding or further complicating surgical candidacy. The patient in question first undergoes treatment of PE to improve hemodynamics and lower the clot burden prior to proceeding with resection of the small bowel. This report emphasizes the utility of the Pulmonary Embolism Response Team to facilitate care when surgical comorbidities require immediate attention as well.

## Introduction

Pulmonary embolism (PE) is a common cause of morbidity and mortality accounting for up to 300,000 deaths in the United States each year [1]. PE is a heterogeneous disease process, and presentation varies widely from asymptomatic to cardiogenic shock and mortality from acute right ventricular (RV) failure. The current gold standard for diagnosis is a computed tomography (CT) pulmonary angiography, which can visualize a thrombus in the pulmonary vessels. Treatment options are based on a patient’s bleeding risk, the severity of the embolus, and hemodynamic status.

Small bowel obstruction is caused by adhesions from prior surgery, hernia, or malignancy [2]. Affected patients show clinical signs of constipation, abdominal pain, and anorexia. Arterial blood gases (ABG) and electrolyte panels may aid in diagnosis by denoting levels consistent with vomiting and dehydration [3]. Abdominal X-rays are used to identify the location of the obstruction while an abdominal CT is used for confirmation. Dilated loops of the small bowel with air-fluid levels and no room for gas are considered diagnostic [4]. Treatment involves bowel rest with nothing by mouth, bowel decompression with a nasogastric (NG) tube, and corrective surgery if there is evidence of a complete block or bowel strangulation.

## Case presentation

**Figure 1:**
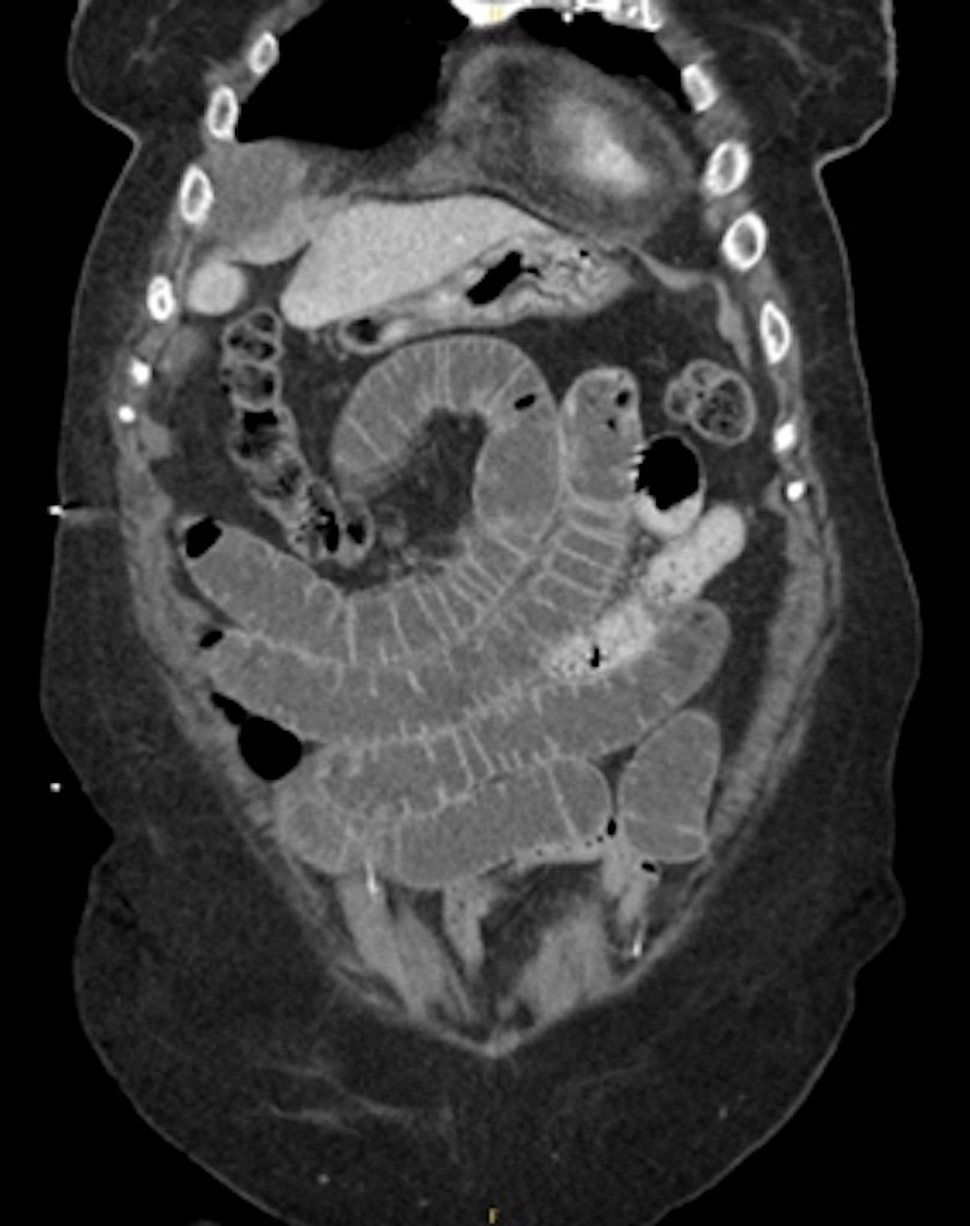
CT abdomen and pelvis showing a small bowel obstruction as evidenced by the inflammatory stranding and tethered loops

**Figure 2:**
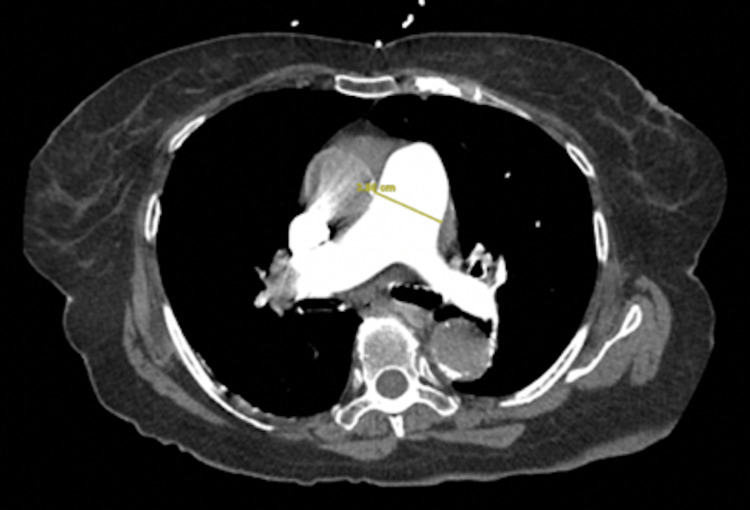
CT angiography of the chest showing pulmonary artery dilation to 3.8 cm

**Figure 3:**
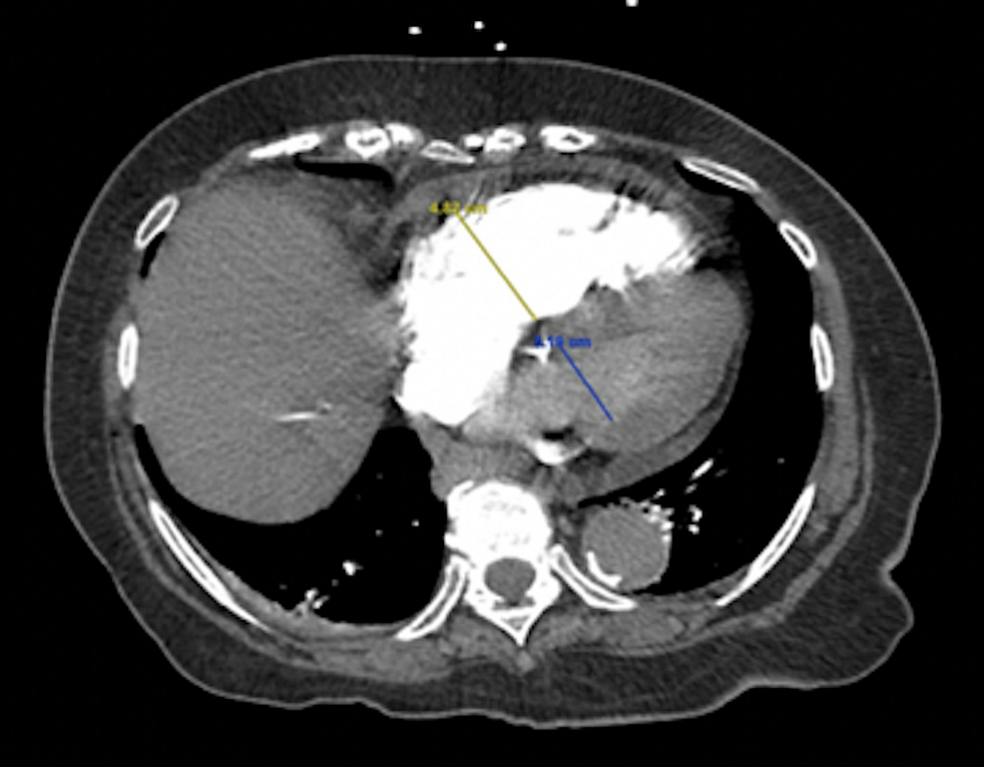
CT angiography of chest showing an increased right ventricular:left ventricular ratio of 1.5

We present an 82-year-old female with a history of hypertension, mild cognitive impairment, and depression. She presented to our institution with two days of abdominal pain and dyspnea. She was tachycardic and hypoxemic with an oxygen saturation of 89% requiring a 2L nasal cannula. The patient had leukocytosis of 19.9 white blood cell count (WBC) x10^9L and acute kidney injury with creatine of 1.39 mg/dL. Additionally, there was evidence of myocardial ischemia with an elevated troponin (0.06 ng/ml) and brain natriuretic peptide (BNP) (448 pg/ml). Her lactate was also elevated (2.2 mmol/L). Subsequent imaging by computed tomography (CT) abdomen and pelvis showed multiple fluid levels characteristic of small bowel obstruction (SBO) with a transition point in the right lower quadrant with inflammatory stranding and tethered loops (Figure 1). A follow-up CT pulmonary angiography visualized bilateral proximal pulmonary embolisms extending into the lower lung bases (Figure 2), with an increased right ventricular:left ventricular (RV/LV) ratio of 1.5 (Figure 3), and a Modified Miller score of 18. A subsequent echocardiogram demonstrated significant physiologic compromise.

**Figure 4:**
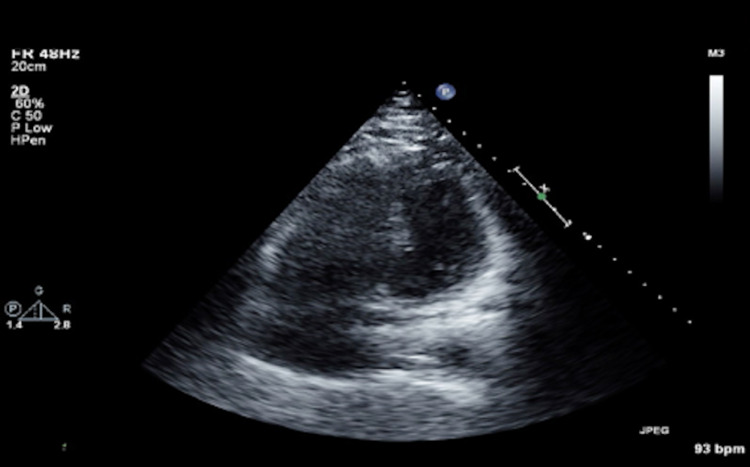
Echocardiography showing an enlarged and hypokinetic right ventricle as a direct result of the submassive pulmonary embolism

**Figure 5:**
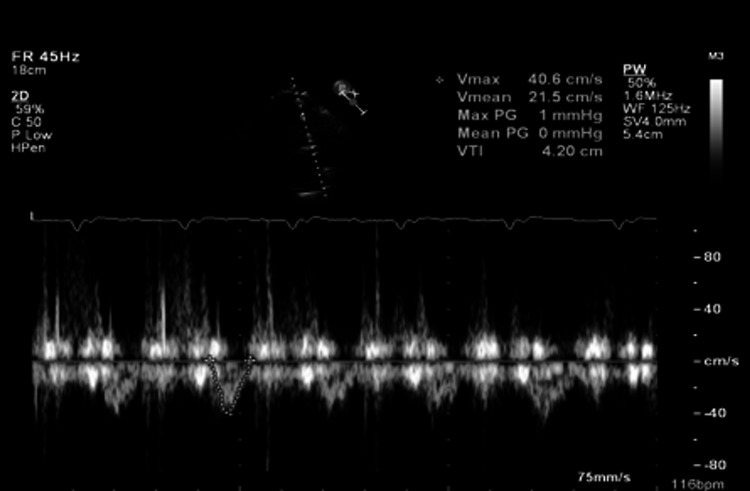
Doppler echocardiography showing a reduced right ventricular outflow tract velocity time integral of 4.20 cm

Specifically, the echo demonstrated an enlarged and hypokinetic right ventricle (RV) (Figure 4) with a reduced right ventricular stroke volume as defined by an outflow tract velocity time integral of 4.20 cm (normal >15) (RVOT VTI) (Figure 5). In summary, the clinical picture was consistent with acute surgical abdomen secondary to small bowel obstruction complicated by a sub-massive intermediate-high risk pulmonary embolism. The patient was felt to be a prohibitive risk for an operation due to concerns for acute decompensation and death from general anesthesia and surgical stress.

After a multidisciplinary Pulmonary Embolism Response Team meeting, a decision was made to take the patient to the cardiac catheterization lab to better understand her hemodynamics. A right heart catheterization demonstrated moderate pulmonary hypertension (pulmonary artery pressures 42/16/25 with a severely reduced cardiac index of 1.8). The decision was made to proceed with aspiration thrombectomy to acutely reduce clot burden and improve hemodynamics. Post-procedure, the pulmonary artery pressures and cardiac output improved to 31/15/17 and 2.3. An inferior vena cava filter was placed given the need to hold intravenous heparin perioperatively. A pulmonary artery catheter was placed to provide real-time hemodynamic monitoring during surgery.

**Figure 6:**
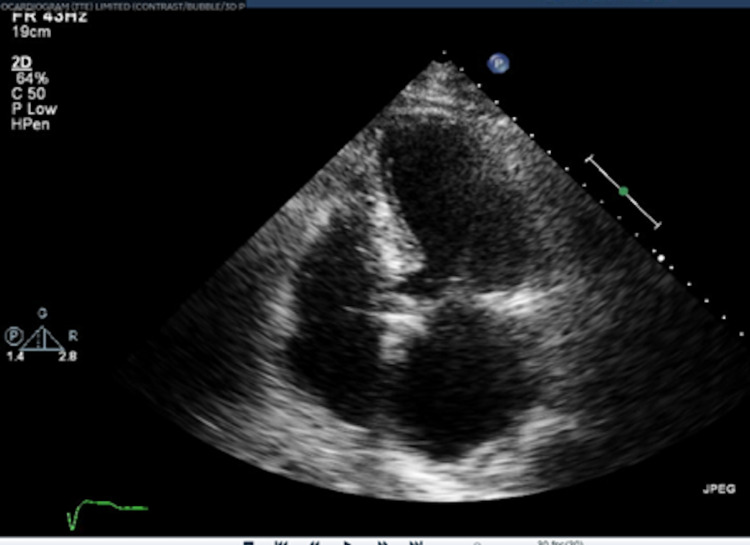
Post-intervention echocardiograph demonstrating improved pulmonary artery pressures and cardiac index of 31/15/17 and 2.3, respectively

Given the marked improvement in hemodynamics, the patient was taken to the operating room after the thrombectomy and underwent successful exploratory laparotomy with segmental resection of the small bowel. The patient was extubated and started on a heparin drip on postoperative day 1. Post-op echocardiogram demonstrated complete recovery of right ventricular hemodynamics (Figure 6). She was transferred to the telemetry floor on postoperative day 2. Follow-up care was scheduled with the Pulmonary Embolism Response Team outpatient clinic in four weeks.

## Discussion

This case highlights the importance of a multidisciplinary approach in the management of acute pulmonary embolism. With the collective input of interventional cardiology, vascular surgery, general surgery, anesthesia, and pulmonology, the decision was made to acutely reduce clot burden to improve right ventricular hemodynamics and proceed with surgery. With the advent of novel endovascular therapies for the management of acute pulmonary embolism, expertise from multiple specialties is optimal.

There is precedence for early surgical intervention in patients with low-intermediate risk pulmonary embolism. A retrospective cohort study of 140 patients who underwent orthopedic surgery found no difference in major bleeding episodes between the PE and non-PE groups when operated on within 30 days of diagnosis (21.1% vs 16%; p=0.463) [5]. Furthermore, there was no significant difference in bleeding risk or duration of hospitalization when controlling for patients with an inferior vena cava filter placement. However, there is a paucity of data in submassive and massive patients such as ours.

Perioperative patients with acute PE are at increased risk for decompensation and death. Due to the geometrical and mechanical differences of the right ventricle (RV) as compared to the left ventricle, the RV is more susceptible to acute failure from sudden pressure overload from a large thrombus [6]. The increase in pressure in the pulmonary vasculature leads to increased afterload and decreased RV stroke volume, ultimately causing a decrease in left ventricular (LV) stroke volume and cardiopulmonary arrest. The use of mechanical thrombectomy devices may offer a solution for subjects who require rapid improvement in hemodynamics. We view invasive hemodynamics and cardiac output as an important metric to gauge hemodynamic response and the likelihood of successful clinical outcomes in the treatment of PE in patients needing urgent surgical intervention.

## Conclusions

Early risk stratification of surgical patients presenting with pulmonary embolism is crucial. This requires a multidisciplinary approach from a broad range of specialists and expert care. Reducing pulmonary artery pressures and improving cardiac output is essential in patients with multiple comorbidities needing advanced care. Access to novel percutaneous interventions serves the best interest of patients and expands options to subjects once thought to be prohibitive surgical candidates. The approach by the Pulmonary Embolism Response Team facilitates excellent time-sensitive care for patients with comorbidities that force clinicians to deviate from standard guidelines to achieve quality clinical outcomes.
